# The Antibacterial and Anti-inflammatory Activity of Chicken Cathelicidin-2 combined with Exogenous Surfactant for the Treatment of Cystic Fibrosis-Associated Pathogens

**DOI:** 10.1038/s41598-017-15558-4

**Published:** 2017-11-14

**Authors:** Brandon J. H. Banaschewski, Brandon Baer, Christina Arsenault, Teah Jazey, Edwin J. A. Veldhuizen, Johan Delport, Tracey Gooyers, James F. Lewis, Henk P. Haagsman, Ruud A. W. Veldhuizen, Cory Yamashita

**Affiliations:** 1 Department of Physiology and Pharmacology, Western University, London, Ontario, Canada; 2 Department of Infectious Diseases and Immunology, Division of Molecular Host Defense, Faculty of Veterinary Medicine, Utrecht University, Utrecht, The Netherlands; 3 London Health Sciences Centre, London, Ontario, Canada; 4 Department of Medicine, Western University, London, Ontario Canada

## Abstract

Cystic fibrosis (CF) is characterized by recurrent airway infections with antibiotic-resistant bacteria and chronic inflammation. Chicken cathelicin-2 (CATH-2) has been shown to exhibit antimicrobial activity against antibiotic-resistant bacteria and to reduce inflammation. In addition, exogenous pulmonary surfactant has been suggested to enhance pulmonary drug delivery. It was hypothesized that CATH-2 when combined with an exogenous surfactant delivery vehicle, bovine lipid extract surfactant (BLES), would exhibit antimicrobial activity against CF-derived bacteria and downregulate inflammation. Twelve strains of CF-pathogens were exposed to BLES+CATH-2 *in vitro* and killing curves were obtained to determine bactericidal activity. Secondly, heat-killed bacteria were administered *in vivo* to elicit a pro-inflammatory response with either a co-administration or delayed administration of BLES+CATH-2 to assess the antimicrobial-independent, anti-inflammatory properties of BLES+CATH-2. CATH-2 alone exhibited potent antimicrobial activity against all clinical strains of antibiotic-resistant bacteria, while BLES+CATH-2 demonstrated a reduction, but significant antimicrobial activity against bacterial isolates. Furthermore, BLES+CATH-2 reduced inflammation *in vivo* when either co-administered with killed bacteria or after delayed administration. The use of a host-defense peptide combined with an exogenous surfactant compound, BLES+CATH-2, is shown to exhibit antimicrobial activity against antibiotic-resistant CF bacterial isolates and reduce inflammation.

## Introduction

Cystic fibrosis (CF) is an autosomal recessive disease caused by defects in the cystic fibrosis transmembrane conductance regulator (CFTR) gene that result in decreased and/or dysfunction of the trans-epithelial protein responsible for chloride and bicarbonate ion transport leading to an increased viscosity of the airway-surface mucosal layer^1^. This altered mucous layer results in chronic airway colonization with bacteria that ultimately leads to a vicious cycle of repeated lower respiratory tract infections, inflammation and tissue remodelling^1,2^. Furthermore, the repeated use of oral, inhaled and systemic antibiotics eventually leads to the development of airway colonization with multi-drug resistant organisms, and represents an independent predictor of adverse clinical outcomes including a more rapid decline in lung function and premature death^3–5^. Ultimately, this complex pro-inflammatory environment induced by chronic bacterial airway infections promotes airway epithelial cell injury and airway remodelling^6–12^ and results in greater challenge from a therapeutic standpoint. Therefore, novel therapies are urgently needed to improve clinical outcomes for patients with CF.

Exogenous cathelicidins are a class of innate host-defense peptides that are currently under investigation for their potential therapeutic use against antibiotic-resistant bacterial infections and have been shown to possess both direct antibacterial activity and immunomodulatory activity. Importantly, based on their multiple mechanism of action, these host defense peptides are notable for their antimicrobial activity against a wide spectrum of bacteria that exhibit resistance to conventional antibiotics and therefore represent an attractive target for therapeutic development^13–16^. Furthermore, an additional capability for cathelicidins to modulate the host inflammatory responses has been identified including alterations of inflammatory cytokine production and immune cell migration^17–25^. Currently, outside of use in topical skin infections, the widespread adoptions of HDPs to treat antibiotic resistant infections remains limited due to constraints surrounding optimization of systemic delivery methods and an ability to achieve sufficient concentrations of peptide at localized sites of infection.

In order to facilitate the pulmonary delivery of therapeutics, exogenous surfactant has been investigated as a potential delivery vehicle due to its ability to improve distribution of therapeutics to peripheral lung regions^26–28^. The use of exogenous surfactant has been extensively investigated as a therapeutic option in patients with acute respiratory distress syndrome and as the standard of care in preterm neonates with surfactant deficiency. Universally, exogenous surfactant administration has been demonstrated to be safe and well tolerated across a spectrum of lung disease, although its benefits as a therapeutic alone in adult populations with ARDS has not been consistently observed^26,28,29^. Previous studies have investigated the use of exogenous pulmonary surfactant with cathelicidin peptides for the treatment of respiratory pathogens^30^. Our group recently demonstrated in a series of ‘proof-of-principle’ experiments, that an approach which combines a chicken cathelicidin, CATH-2, with a commercially available exogenous surfactant preparation, bovine lipid-extract surfactant (BLES), maintains excellent surfactant spreading properties, antimicrobial activity, and is well tolerated when administered intratracheally to naïve mice^31^.

The objective of the current study was to assess specific antimicrobial and/or anti-inflammatory properties of a BLES+CATH-2 preparation, as a novel therapeutic approach to treat CF-related lung infections. It was hypothesized that BLES+CATH-2 would exhibit potent bactericidal activity against bacterial isolates obtained from CF patients and, additionally, could reduce inflammation associated with bacterial killing *in vivo*.

## Results

### Resistance Patterns of Clinical CF Bacterial Isolates

Clinical characteristics and *in vitro* antibiotic-resistance patterns of bacterial isolates obtained from CF patients were obtained from the London Health Science Centre Clinical Microbiology Laboratory and are shown in Table 1. Patients ranged between 24 and 57 years of age. Disease severity as measured by lung function was variable among patients, with the predicted forced expiratory volume in one second (FEV1) ranging between 28% and 87% predicted. Six out of nine patients were colonized with at least one strain of *P. aeruginosa*, 3 out of the 9 patients were infected with *S. aureus*, and one patient was infected with *A. xylosoxidans*. Of the eight strains of *P. aeruginosa* isolated, half were identified as a ‘mucoid’ phenotype. Of all bacteria isolated, seven isolates were resistant to at least two conventional antibiotics while two strains were resistant to three or more antibiotics.

**Table 1:** Demographics and resistance profiles of clinical isolates utilized in the study.

Patient No.	Isolate No.	Sex (M/F)	Age (years)	Weight (kg)	BMI (kgm^2)	FEV1 Pred	Bacteria	Resistance Profiles
1	1	F	57	60.6	22.5	39	*Pseudomonas aeruginosa*	NR
2	2	M	24	72	25.3	50	*Staphylococcus aureus*	Clindamycin, Erythromycin R
3	3	M	50	57	19.3	43	*Achromobacter xylosoxidans*	Gentamicin, Meropenem, Tobramycin R; Imipenem, Ciprofloxacin I
4	4A	F	35	62	22.4	87	*Staphylococcus aureus*	NR
	4B						*Pseudomonas aeruginosa* (muc)	NR
	4C						*Pseudomonas aeruginosa*	Tobramycin, Gentamicin R
5	5	F	44	59	22	73	*Pseudomonas aeruginosa* (muc)	Amikacin, Gentamicin, Meropenem, Ciprofloxacin, Tobramycin R
6	6	F	40	57	20.9	52	*Pseudomonas aeruginosa* (muc)	Gentamicin, Tobramycin R
7	7	M	37	52.5	19.3	28	*Pseudomonas aeruginosa* (muc)	NR
8	8	M	27	62	18.7	48	*Staphylococcus aureus*	Clindamycin, Erythromycin, Cloxacillin R
9	9A	F	31	40.5	18.2	55	*Pseudomonas aeruginosa (muc)*	NR
	9B						*Pseudomonas aeruginosa*	Meropenem R; Ciprofloxacin, Gentamicin I

### Bactericidal Activity of BLES+CATH-2 Against Clinical Isolates

**Figure 1:**
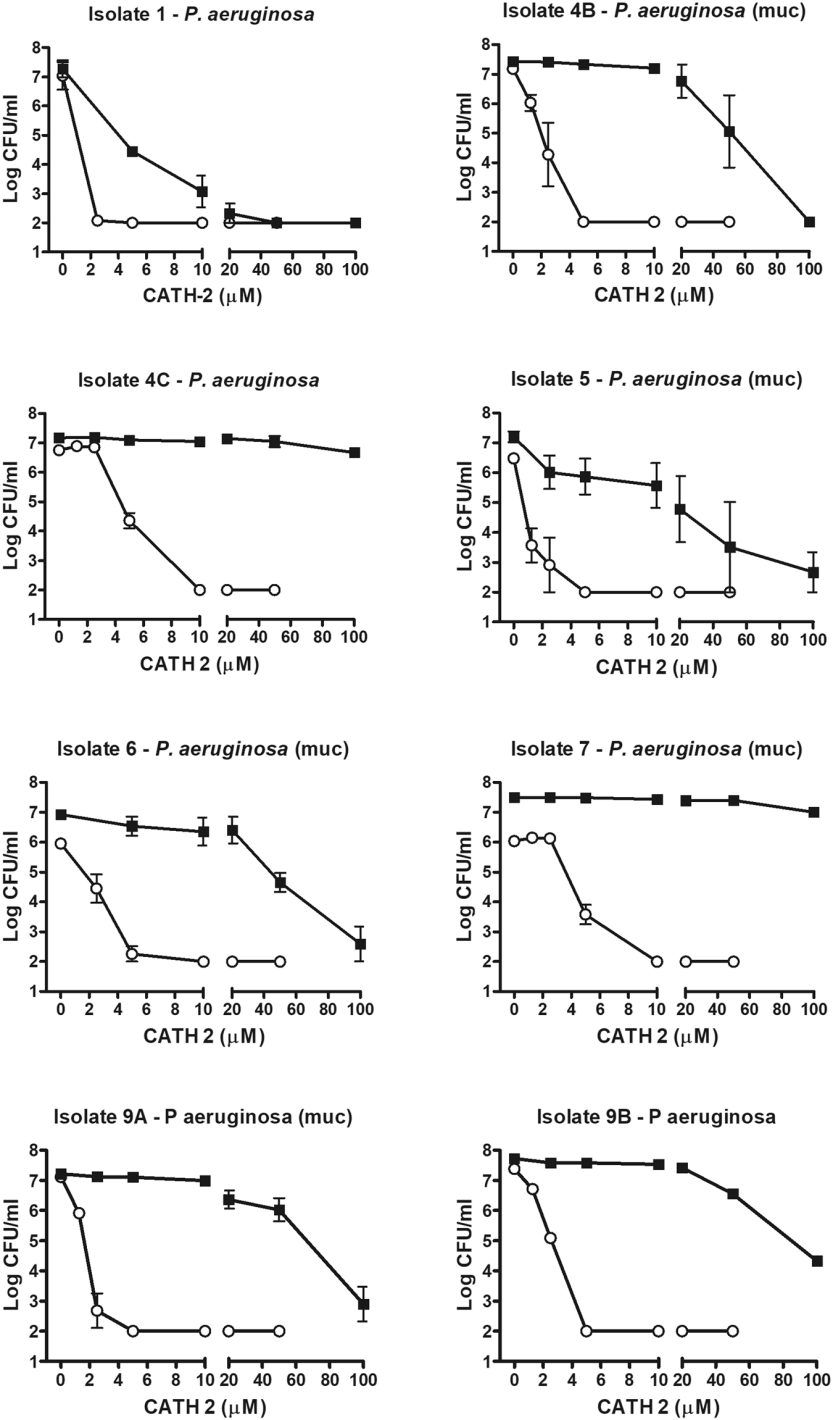
Colony count assays of CATH-2 and BLES+CATH-2 against *P. aeruginosa* isolates. The bacteria were suspended in MHB, and then treated with various concentrations of CATH-2 (open circles) or CATH-2 suspended in 10 mg/ml phospholipid of BLES (solid squares). A value of Log CFU/ml of 2 represents the detection limit of the analysis.

After *in vitro* incubation of bacterial isolates with either CATH-2 or BLES+CATH-2, it was observed that CATH-2 alone exhibited potent bactericidal activity against all *P. aeruginosa* isolates, regardless of *in vitro* antibiotic-resistance profiles, with the Minimum bactericidal concentration (MBC) ranging between 2.5–10 µM of CATH-2 (Fig. 1). BLES+CATH-2 exhibited greater bactericidal variability against *P. aeruginosa* isolates, with bacterial activity ranging between a 3-log reduction in bacterial growth to complete bacterial killing. Overall, BLES+CATH-2 MBC values were typically 100 µM, and as low as 50 µM against selected CF-derived isolates (Fig. 1).

**Figure 2:**
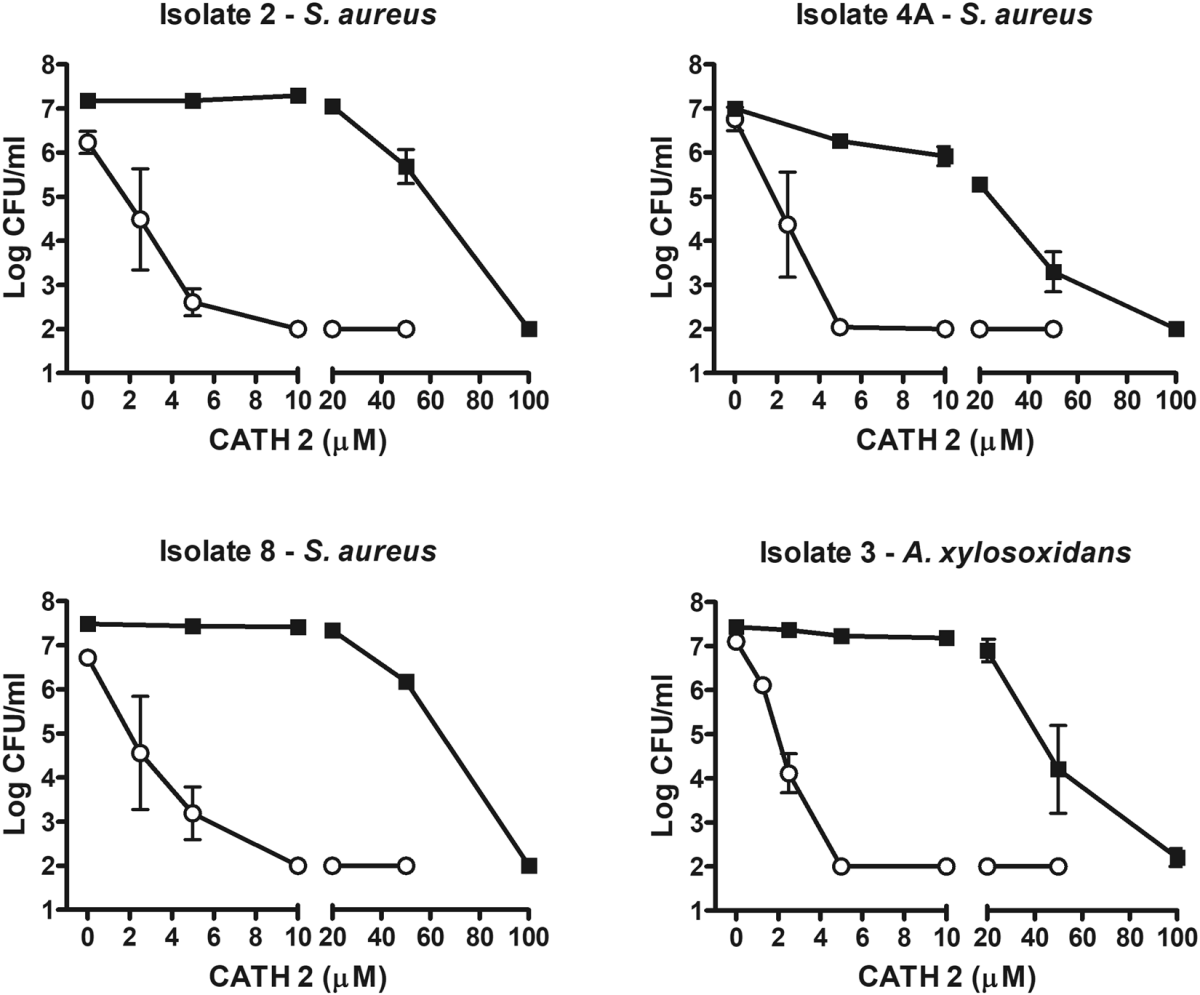
Colony Count assays of CATH-2 and BLES+CATH-2 against three *S. aureus* isolates and one *A. xylosoxidans* isolate. The bacteria were suspended in MHB, and then treated with various concentrations of CATH-2 (open circles) or CATH-2 suspended in 10 mg/ml phospholipid of BLES (solid squares). A value of Log CFU/ml of 2 represents the detection limit of the analysis.

Similar to bactericidal activity against *P. aeruginosa*, CATH-2 exhibited potent antimicrobial activity against all three strains of *S. aureus* tested. CATH-2 exhibited bactericidal activity against all *S. aureus* isolates investigated, with MBC values between 5–10 µM concentrations for all strains investigated (Fig. 2). BLES+CATH-2 showed consistent bactericidal activity, and achieved MBC values at 100 µM against all *S. aureus* isolates tested. Bactericidal activity of both CATH-2 and BLES+CATH-2 against *A. xylosoxidans* was similar to the killing profiles observed with *P. aeruginosa* and *S. aureus* (Fig. 2).

### Anti-inflammatory effects of BLES+CATH-2 *in vivo*

To investigate the potential anti-inflammatory effects of BLES+CATH-2, we utilized a model whereby heat-killed bacteria were instilled intratracheally in mice to elicit a proinflammatory response. Through this technique, the antimicrobial-independent anti-inflammatory properties of BLES+CATH-2 could be more accurately assessed. One antibiotic-resistant strain of *P. aeruginosa* (obtained from Patient 6) and one antibiotic-resistant strain of *S. aureus* (obtained from Patient 4) was chosen randomly from CF patient samples (see Table 1). Heat-killed isolates were incubated with either CATH-2 or BLES+CATH-2 *ex-vivo*, prior to intratracheal instillation, to specifically evaluate bactericidal independent anti-inflammatory effects. Initially, lung function parameters (as measured using a Flexivent©), and protein values in the lavage were analyzed to determine potential effects of BLES+CATH-2 on the development of lung injury. These results revealed no significant differences in lung compliance, elastance or protein values among the 5 experimental groups (Table 2).

**Table 2:** Protein content obtained from BALF after whole lung lavage, and quasi-static compliance and elastance as measured by FlexiVent.

Treatment	Protein Content (mg/kg BW)	Quasi-Static Compliance (ml/cmH_2_O)	Quasi-Static Elastance (cmH_2_O/ml)
Control	21.60 ± 3.0	0.0777 ± 0.016	13.278 ± 2.70
*P. aeruginosa*			
Heat-killed	20.56 ± 9.7	0.0718 ± 0.0080	14.077 ± 1.76
Heat+CATH-2	19.44 ± 5.9	0.0755 ± 0.0069	13.347 ± 1.34
Heat+(BLES+CATH-2)	17.58 ± 4.9	0.0801 ± 0.0064	12.558 ± 1.07
*S. aureus*			
Heat-killed	20.79 ± 5.2	0.0754 ± 0.0132	13.700 ± 3.02
Heat+CATH-2	20.98 ± 7.8	0.0736 ± 0.0080	13.742 ± 1.64
Heat+(BLES+CATH-2)	18.39 ± 8.0	0.0712 ± 0.0071	14.156 ± 1.46

**Figure 3:**
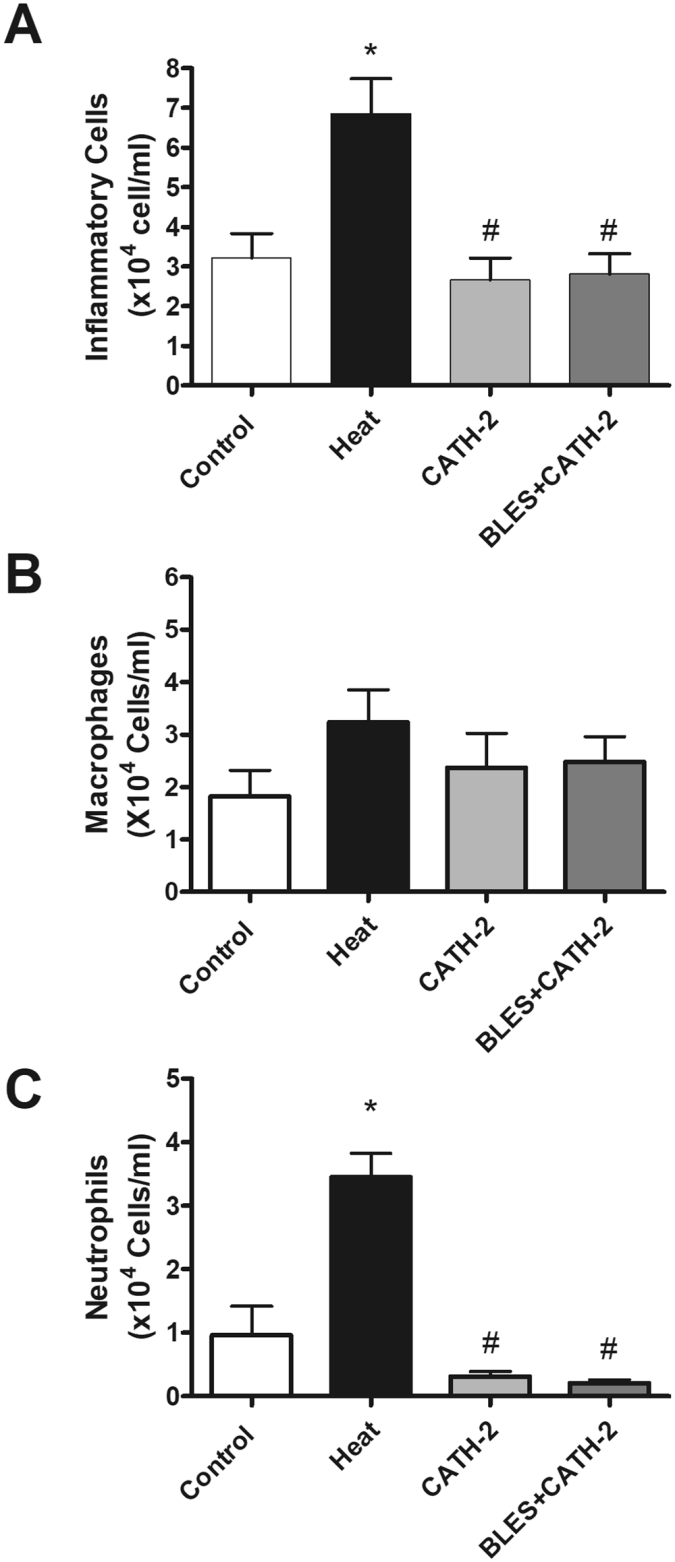
Inflammatory cells obtained from BALF of control animals and animals administered heat-killed *P. aeruginosa*, either alone, or co-administered with CATH-2 or with BLES+CATH-2, six hours after instillation. (**A**) Total cell counts, (**B**) Macrophage, and (**C**) neutrophil *p ≤ 0.05 vs. control, ^#^p < 0.001 vs. heat-killed *P. aeruginosa*.

**Figure 4:**
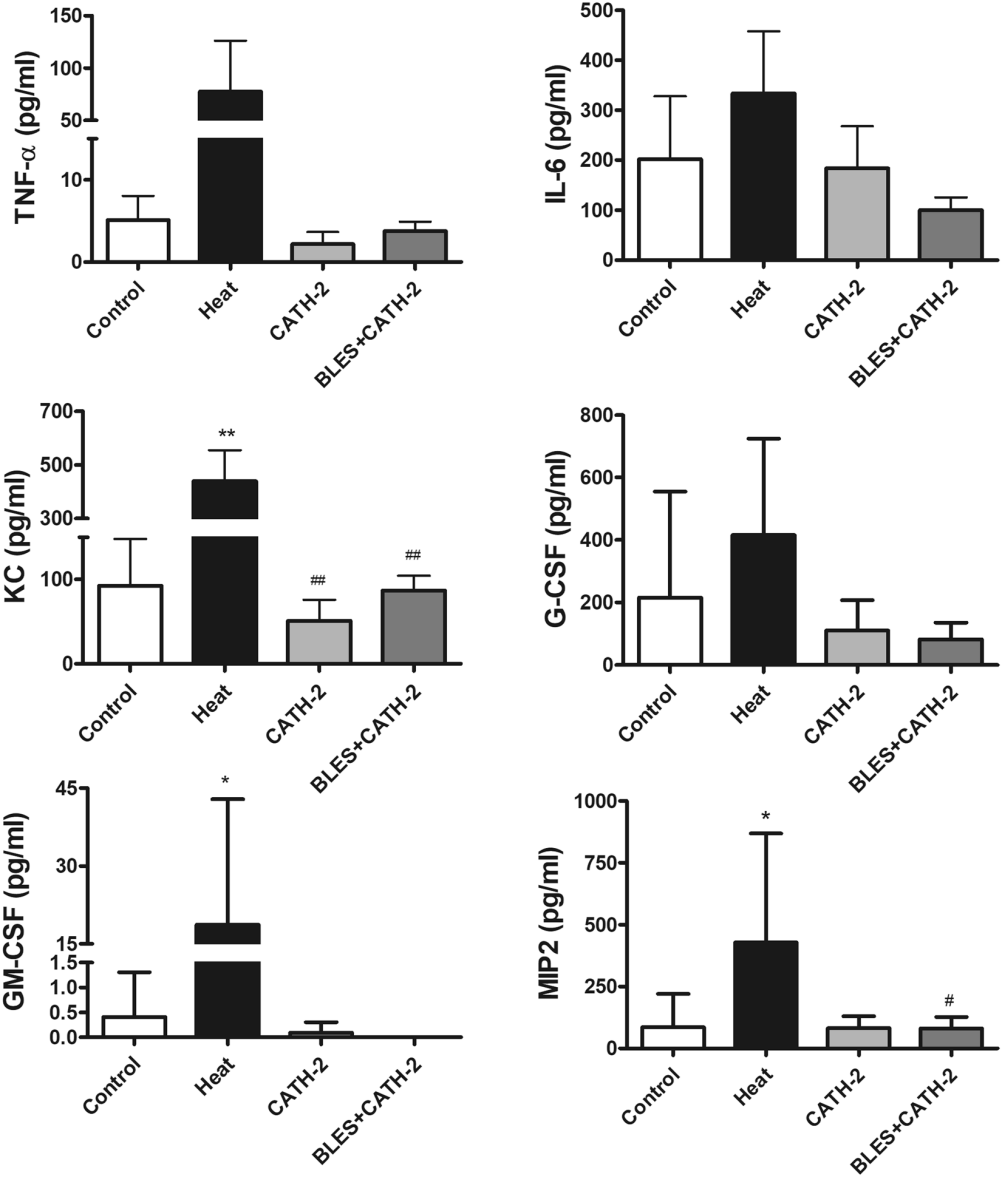
Cytokine content in the BALF obtained from animals six hours after administration of killed *P. aeruginosa*, either alone, or co-administered with CATH-2 or BLES+CATH-2. *p ≤ 0.05 vs. control, **p ≤ 0.01 vs. control, ^##^p ≤ 0.01 vs. heat-killed.

A significant increase in total cell counts in the BALF was observed in animals that received intratracheally-administered heat-killed *P. aeruginosa* compared to the group of animals receiving saline only. On the other hand, incubation of heat-killed *P. aeruginosa* with either CATH-2 or BLES+CATH-2 prior to instillation reduced lavage cell counts similar to levels observed with saline control instillation (Fig. 3A). Differential cell counts demonstrated a significant increase in neutrophils in animals receiving heat-killed *P. aeruginosa* compared to saline control animals, whereas macrophages were not significantly different (Fig. 3B and C). Animals instilled with heat-killed bacteria supplemented with CATH-2 or BLES+CATH-2 had macrophage and neutrophil counts that were similar to saline control animals (p > 0.05). A similar pattern of change was observed for concentration of BALF inflammatory cytokines with heat-killed *P. aeruginosa* eliciting a significant increase in KC, MIP2, and GM-CSF compared to saline controls, with not significant increases observed in TNF-α and IL-6 (Fig. 4). Likewise, animals receiving heat-killed bacteria supplemented with CATH-2, either alone or in combination with BLES, did not result in a significant increase of any of the BALF inflammatory cytokines measured and was not statistically different from saline controls.

**Figure 5:**
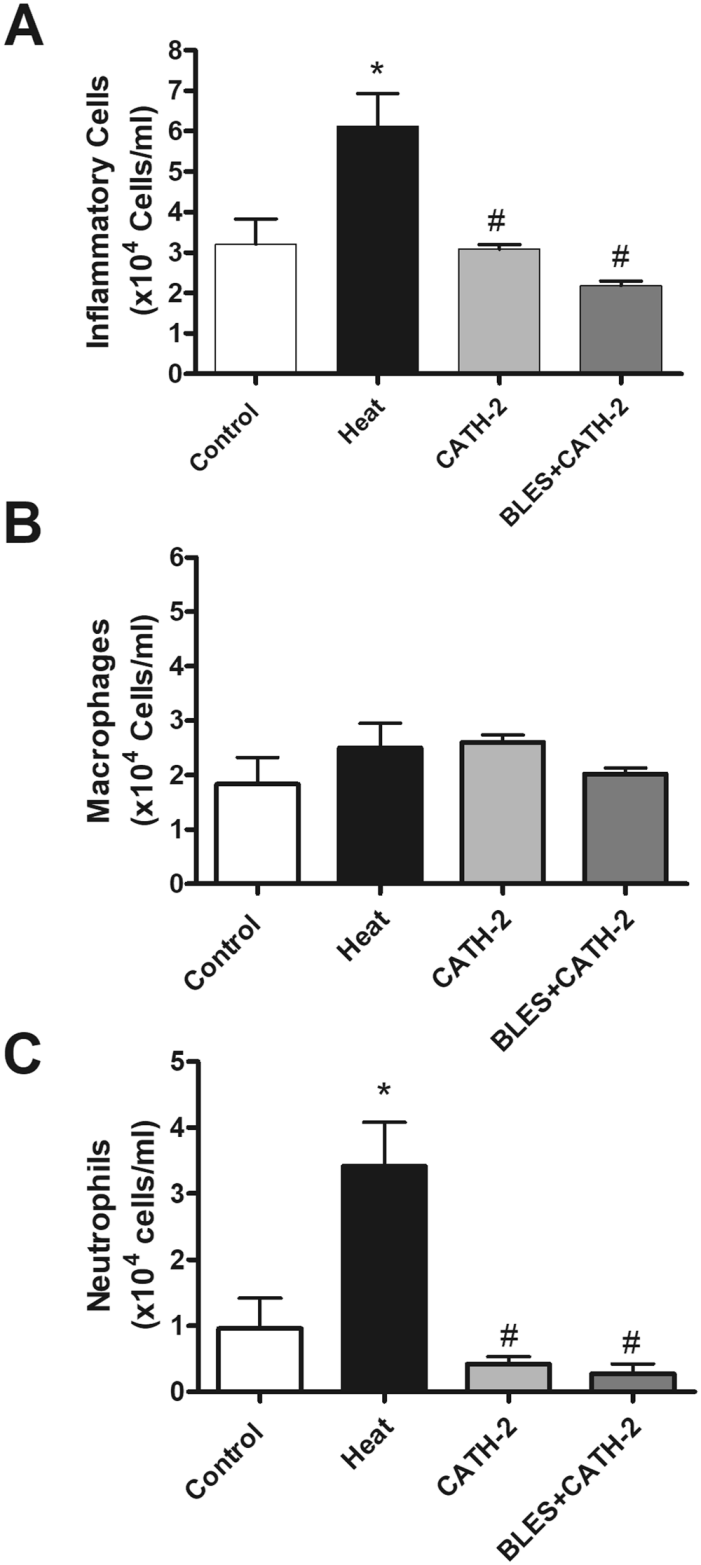
Inflammatory cells obtained from BALF of control animals and animals administered heat-killed *S. aureus*, either alone, with CATH-2 or with BLES+CATH-2 six hours after instillation. (**A**) Total cell counts, (**B**) Macrophage, and (**C**) neutrophil *p < 0.05 vs. control, ^#^p ≤ 0.001 vs. heat-killed *S. aureus*.

**Figure 6:**
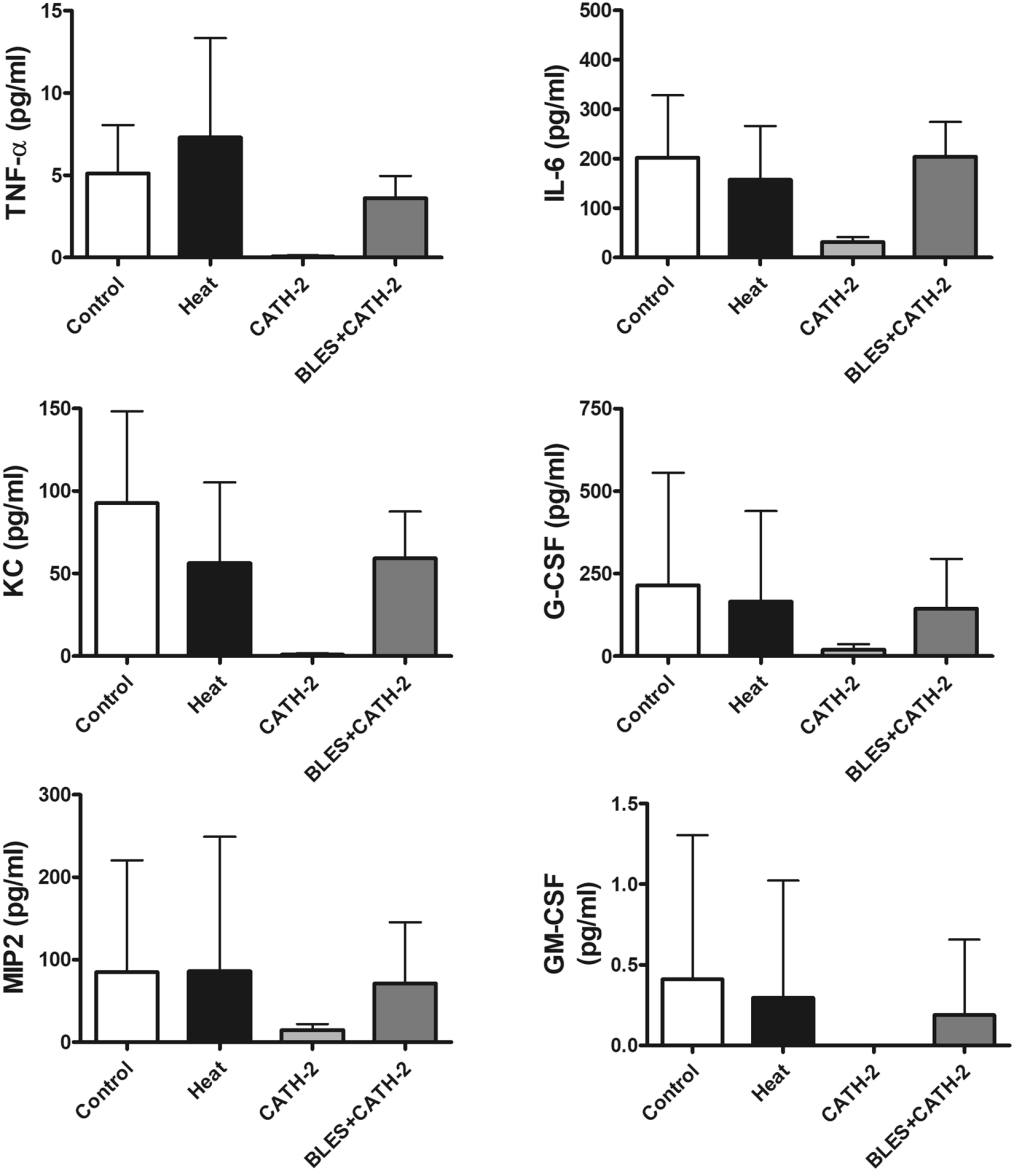
Cytokine content in the BALF obtained from animals six hours after administration of killed *S. aureus* either alone, with CATH-2 or with BLES+CATH-2.

The results for the inflammatory responses observed with a killed *S. aureus* isolate are shown in Figs 5 and 6. Similar to observations made for *P. aeruginosa*, incubation of *S. aureus* with CATH-2 or BLES+CATH-2 resulted in a significant reduction in the total cell counts recovered from the BALF compared to heat-killed bacteria alone (Fig. 5A). Differential cell counts revealed that heat-killed *S. aureus* resulted in significantly higher neutrophil counts compared to other groups (Fig. 5C). Despite the differences observed in total cell counts and differential cell counts, no effect on pro-inflammatory cytokines was observed for instillation of heat killed *S. aureus*, and furthermore, supplementation with CATH-2 or BLES+CATH-2 did also not lead to any significant differences in cytokine counts although concentrations were generally lower in the CATH-2 group (Fig. 6).

### Effect of delayed BLES+CATH-2 administration after induction of inflammation with dead bacteria

**Figure 7:**
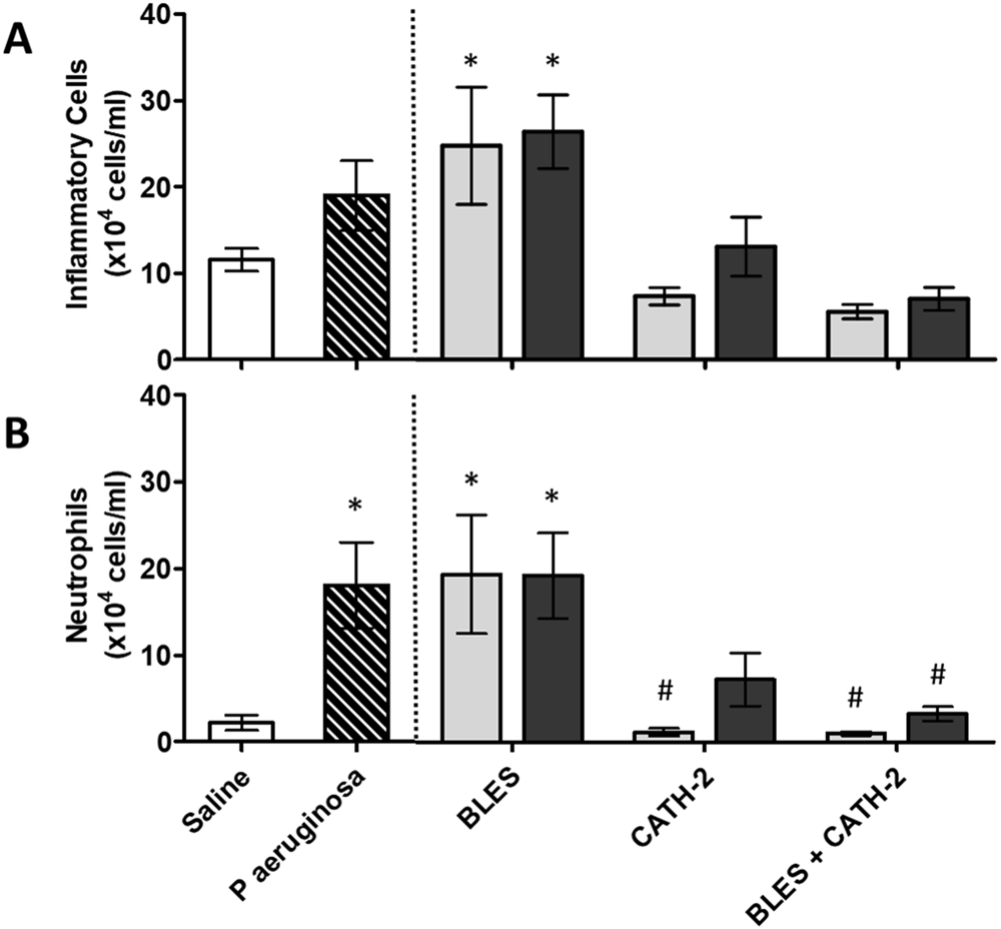
The total number of inflammatory cells (**A**) and Neutrophils (**B**) per mL of lung lavage samples collected from mice in the different experimental groups four hours after instillation of killed bacteria. The negative (Saline) and positive (*P. aeruginosa*) controls are represented as the two bars to the left of the dotted line. The bars to the right of the dotted line represent the data for the 6 groups that had their treatments either co-administered with the dead bacteria, or 15 minutes after the administration of dead bacteria, for three treatments: BLES, CATH-2, and BLES+CATH-2. *p ≤ 0.05 vs. Saline, ^#^P < 0.05 vs. *P. aeruginosa*.

The second *in vivo* experiment assessed whether BLES+CATH-2 could reduce the inflammatory response induced by heat-killed *P. aeruginosa*, if administered after a 15-minute delay subsequent to the intratracheal delivery of the dead bacteria. Similar to what was observed in the prior experiment, instillation of a heat-killed laboratory strain of *P. aeruginosa* resulted in an elevated number of inflammatory cells, the majority of which were neutrophils and this effect was mitigated by co-administration of CATH-2 or BLES+CATH-2 (Fig. 7A,B). Subsequently, the delayed administration of CATH-2 alone exhibited values of BAL neutrophil counts that were not different from the heat-killed *P. aeruginosa* group, however, the delayed administration of BLES+CATH-2 resulted in total cell count values that were significantly lower than that of the heat-killed *P. aeruginosa* group (Fig. 7B). Administration of BLES alone had no effect on inflammatory cell influx.

**Figure 8:**
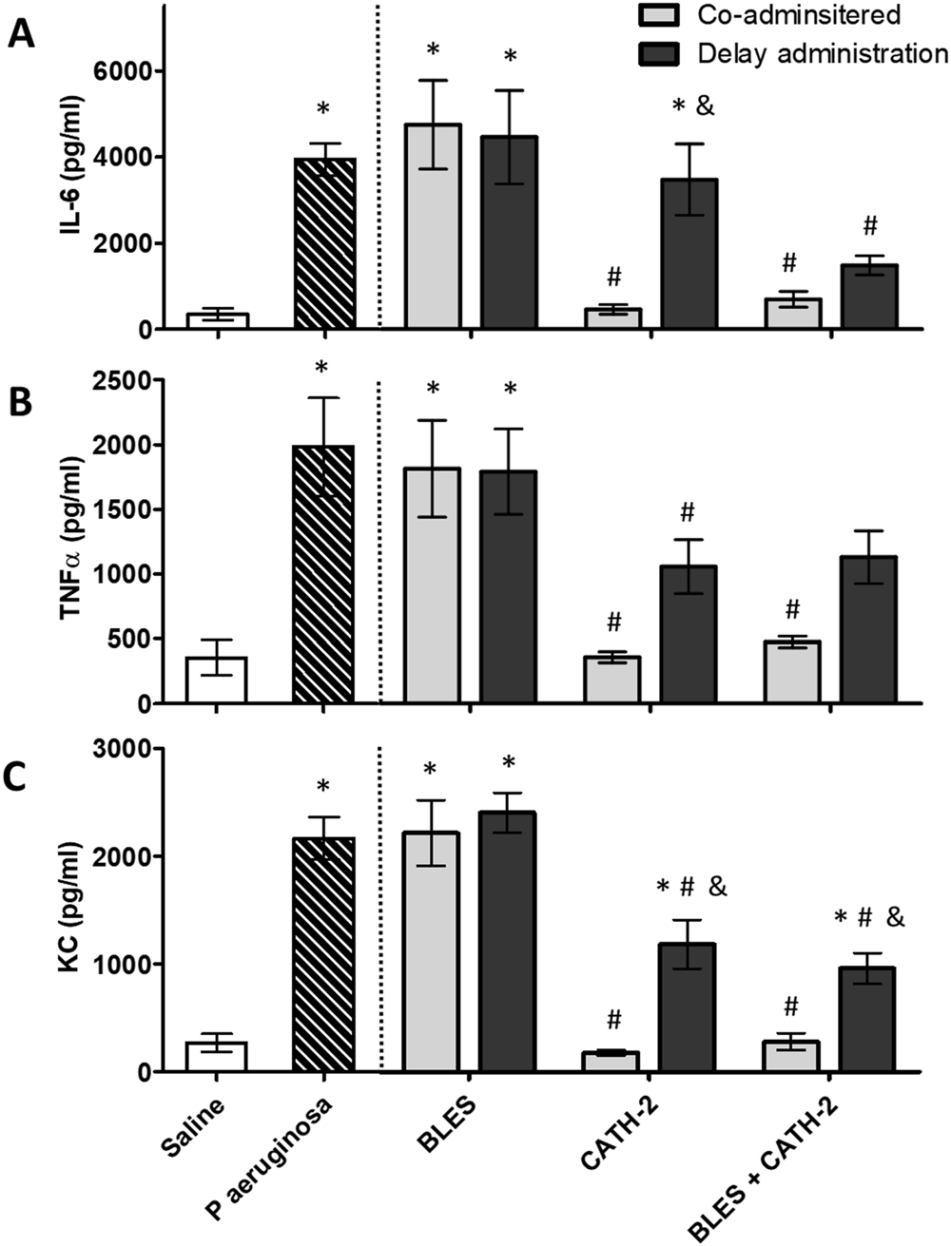
The concentration of inflammatory mediators, IL-6 (**A**), TNFα, (**B**) and KC (**C**) in lung lavage samples collected from mice in the different experimental groups four hours after instillation of killed bacteria. The negative (Saline) and positive (*P. aeruginosa*) controls are represented as the two bars to the left of the dotted line. The bars to the right of the dotted line represent the data for the 6 groups that had their treatments either co-administered with the dead bacteria, or 15 minutes after the administration of dead bacteria, for three treatments: BLES, CATH-2, and BLES+CATH-2. *P < 0.05 vs. Saline, ^#^P < 0.05 vs. *P. aeruginosa*, ^&^p ≤ 0.05 vs. Co-administration values for the same treatment.

Concentrations of three inflammatory mediators in the eight experimental groups are shown in Fig. 8. Heat killed *P. aeruginosa* caused a significant increase in lavage concentrations of IL-6, TNF-α and KC compared to saline control. Co-administration of CATH-2 or BLES+CATH-2 resulted in a complete mitigation of the cytokine response resulting in concentrations not significantly different from saline control values. Delayed administration of CATH-2 resulted in values that were slightly higher than those of co-administration with the difference reaching statistical significance for IL-6 and KC. Lavage concentrations of the three cytokines after delayed administration of BLES+CATH-2 reached values in between those of the positive, *P. aeruginosa*, and negative, Saline, control groups with only KC being significantly different from co-administration values. Administration of BLES without CATH-2 had no effect on lavage cytokine concentration.

## Discussion

In the current study, the antimicrobial and anti-inflammatory effects of BLES+CATH-2, a chicken cathelicidin suspended in a clinical exogenous surfactant preparation, BLES, were evaluated for therapeutic potential as a novel treatment for antibiotic-resistant bacterial lung infections. The data presented in the current manuscript demonstrate that BLES+CATH-2 exhibits bactericidal activity against an array of antibiotic-resistant bacterial isolates obtained from patients with cystic fibrosis. Furthermore, BLES+CATH-2 was demonstrated to mitigate the recruitment of inflammatory cells and modulate proinflammatory cytokine production when co-administered with heat-killed pathogens, and had the ability to mitigate inflammatory cell recruitment when administered after initiation of inflammation. This data provides evidence for a potential therapy targeting antibiotic-resistant infections and associated inflammation using a combined host defense peptide and exogenous surfactant delivery vehicle.

The first goal of this study was to identify the bactericidal properties of BLES+CATH-2 against multiple cystic fibrosis-associated antibiotic-resistant bacteria. *P. aeruginosa* represents one of the most prevalent bacteria found in CF patients, while *S. aureus* is the most common bacteria in patients under the age of 18^9,32–36^. Due to the frequent use of conventional antibiotics used to treat these pathogens both on an acute and chronic basis, the prevalence of antibiotic resistance among these strains has been reported to be as high as 22.6% for *S. aureus* and 42.7% for *P. aeruginosa* among patients with CF^37,38^. Consistent with this information, the specific strains of *P. aeruginosa, S. aureus*, and *A. xylosoxidans* isolated from CF patient sputum samples in our study, displayed a wide range of antibiotic resistance patterns. Although the antimicrobial activity of BLES+CATH-2 against these clinical strains was reduced compared to the CATH-2 peptide alone, BLES+CATH-2 exhibited significant antimicrobial activity at higher CATH-2 concentrations, with the compound able to reduce eight out of twelve bacterial isolates to below detectable levels after three-hour incubations. For the remaining four bacteria tested, BLES+CATH-2 was able to reduce bacteria by three log values for two of the isolated bacteria. This data expands upon work published previously by our research group which demonstrated the efficacy of BLES+CATH-2 against laboratory strains of *P. aeruginosa* or *S. aureus* ^31^. Based on previous findings that a 100 µM concentration of BLES+CATH-2 was well tolerated in naïve mice, this concentration was chosen for subsequent *in vivo* studies to assess the specific antibiotic-independent anti-inflammatory properties of BLES+CATH-2.

To test the ability of CATH-2, and BLES+CATH-2 to modulate inflammatory responses induced by bacterial products we utilized a model in which killed CF isolates were administered intratracheally to naïve mice to induce an inflammatory response. This experimental technique was utilized to assess the immunomodulatory properties of CATH-2 and BLES+CATH-2. The data obtained provided evidence that CATH-2, and BLES+CATH-2, can down-regulate inflammatory responses induced by killed bacteria including reductions in proinflammatory mediators including IL-6, TNF-α and KC. This data builds on previous observations utilizing *E.coli* or a laboratory strain of *P. aeruginosa* in which CATH-2 killed bacteria resulted in a downregulated inflammatory response as compared to antibiotic or heat killed bacteria^39,40^. Specifically, our data extended these observations by demonstrating this anti-inflammatory effect utilizing two clinical strains of bacteria, including a Gram-positive and a Gram-negative isolate. In addition, our data demonstrated that BLES+CATH-2 maintained this anti-inflammatory activity previously shown for just CATH-2 by itself. Overall, this ability of BLES+CATH-2 to not only kill clinically isolated antibiotic resistant bacteria, but to also diminish the inflammatory response induced by bacterial products after killing may provide a unique multi-functional therapeutic approach that would be of high therapeutic potential particularly in the context of CF^1,2,12,32,41^.

Interestingly, whereas heat-killed *P. aeruginosa* induced a significant pro-inflammatory cytokine response at our 6 hour timepoint following instillation, administration of heat-killed *S. aureus* did not lead to this increased inflammatory cytokine production, despite a significant increase in total cells and neutrophils in the lavage fluid. These results appear to contradict other studies which demonstrated a significant increase in TNF-α production after exposure of alveolar macrophages to heat-killed *S. aureus in vitro* ^42^. It is possible that different methods of heat-killing may have altered the immunogenic by-products of *S. aureus*, which led to a reduction in cytokine induction. Alternatively, the levels of inflammatory cytokine in the BALF my exhibit temporal variation and therefore could have returned to baseline levels at the point of assessment (i.e. four hours after instillation), despite persistence of elevated cell counts.

The use of exogenous surfactant preparations as a vehicle to delivery therapeutics to the lung has been previously explored for a variety of therapies including conventional antibiotics^27–29,43–46^. Advantages of this approach over other techniques include the ability to increase therapeutic concentrations deposited in the lung and, based on the innate spreading properties of surfactant, a more uniform distribution throughout the lung^26,47^. Furthermore, based on its inherent biophysical properties, exogenous surfactant has the ability to open closed lung units and blocked airways which may be of particular relevance in the setting of CF and viscoid airway secretions. Previous work published by our group demonstrated that the addition of BLES as a vehicle for host-defense peptides, to some extent, mitigated the antimicrobial activity of host-defense peptides compared to the innate antimicrobial activity of the peptide alone. Therefore, the ability of BLES+CATH-2 to reduce inflammation compared to CATH—2 alone after an established inflammatory insult, would provide significant rationale for this combined approach utilizing BLES as a drug delivery vehicle.

Consistent with the above conclusion, the current study demonstrated that the addition of BLES to CATH-2, compared to the delivery of intratracheal CATH-2 alone demonstrated superior outcomes such as IL-6 concentrations in the BAL, when delivered after the administration of dead bacteria with respect to reduction in inflammation. We speculate that this effect is likely due to the enhanced distribution of CATH-2 obtained with BLES, as the administration of BLES alone failed to exhibit any independent anti-inflammatory properties. Another explanation might be that CATH-2, associated with surfactant, is released slowly resulting in a more sustained effect of the peptide. It is therefore notable that the suspension in surfactant is an important aspect of utilizing CATH-2 for pulmonary infections. In addition to the advantages list above in terms of distribution and local concentrations, this method bypasses systemic administration and associated side effects^48,49^. Further support of the importance of targeted delivery for CATH-2 comes from the clinical data that indicates that the most promising utilization of host defense peptides for infection comes from its use for skin wound healing in which a peptide containing cream can be directly applied to the infection. This localized administration appears especially important for antimicrobial purposes but may also be important for immunomodulation effects of the peptides.

The antimicrobial mechanisms of action for CATH-2 and other cathelicidins has been reported extensively^13,22,23,50^. The pathways involved in the anti-inflammatory properties of these peptides requires further study, and broadly encompass a number of both pro- and anti-inflammatory processes, such as cytokine production, recruitment of immune cells and alterations to apoptotic pathways^51–53^. It has been shown that CATH-2, and other cathelicidin peptides, have the ability to bind directly to LPS and LTA, thereby limiting downstream activation of inflammatory cytokines via toll-like receptor signalling cascades^54–56^. The anti-inflammatory effects of cathelicidins appear to be independent on cell type, as they have been demonstrated *in vitro* to effect inflammatory cytokine production from multiple different cell lineages^57^. In our study, the anti-inflammatory effects were also observed when BLES+CATH-2 was administered 15 minutes after killed bacteria were administered intratracheally, suggesting that preventing toll-like receptor activation may not be the sole mechanism, and that some other immunomodulatory activity of the cathelicidin, such as alterations of cellular apoptosis, is responsible for these observations. Further support for this comes from data showing that PMAP-23, a porcine cathelicidin with low LPS affinity, maintained some anti-inflammatory properties against heat-killed *P. aeruginosa*. Thus, although binding of LPS likely contributes to the anti-inflammatory properties of CATH-2, other properties certainly will contribute and require further study.

From a clinical perspective, the findings of the current study are of relevance not only to CF associated lung infections, but may also have an impact on other infections complicated by resistant bacteria or difficult to treat bacteria like tuberculosis, ventilator-associated pneumonia, and severe community-acquired pneumonia. In each of these circumstances, clinical outcomes can be directly linked to the presence or absence of antibiotic-resistant organisms^58,59^. Despite a looming global pandemic stemming from antimicrobial resistance, the response on the part of the pharmaceutical industry to develop novel antibiotics has been underwhelming, and therefore a high premium has been placed on the development to novel, antimicrobial approaches. Although our pilot studies will require much more in-depth investigation prior to clinical application, our experiments will serve as a critical framework by which further host-defense peptides and exogenous surfactant preparations can be evaluated. Unfortunately, at the current time, there are limited options in individuals with CF who harbour multidrug resistant pathogens, however a novel therapy such as BLES+CATH-2 may provide an alternate treatment as a means of delaying disease progression and CF associated morbidity.

Despite the insight into the properties of BLES+CATH-2 that our study provides, there are several limitations that need to be considered. First, the dosing in our anti-inflammatory experiments was 20 µM of CATH-2 and 100 µM CATH-2 when suspended in BLES. This strategy was employed as BLES was shown to mitigate direct bacterial killing to an extent whereby these two concentrations are, approximately, similar in bactericidal efficacy; however, the dosing effect on anti-inflammatory properties could be different. A limitation of the second *in vivo* study was that only a 15-minute delay was assessed in treatment subsequent to the administration of killed bacteria, and therefore, future studies examining the specific anti-inflammatory effects of BLES+CATH-2 after a prolonged latency or in models of polymicrobial, chronic bacterial lung infections would be of high interest. Finally, our study investigated the independent bactericidal and anti-inflammatory effect of BLES+CATH-2 in separate experimental approaches. Future studies should investigate both phenomenon in *in vivo* models of bacterial infection.

In conclusion, it is demonstrated that the chicken cathelicidin, CATH-2, in combination with an exogenous surfactant, BLES, possesses both bactericidal activity against clinically-derived CF pathogens, and can furthermore downregulate inflammation through antimicrobial-independent mechanisms by silencing the inflammatory response induced by killed bacteria. Taken together, the ability to kill multi-drug resistant bacteria while being able to modulate the hyper-inflammatory environment induced by these pathogens provides strong evidence for the potential of BLES+CATH-2 as a therapeutic for the management of respiratory infections associated with CF and other pulmonary bacterial infection.

## Methods

All experimental protocols were approved by the Lawson Health Research Institute (London, Ontario, Canada).

### Surfactant/Peptide Compounds

Chicken cathelicidin CATH-2 was synthesized using Fmoc solid-phase synthesis as described previously^60^. All peptides were purified to a minimum purity of 95% by reverse phase high-performance liquid chromatography prior to biological testing. The peptide was then suspended in non-buffered sterile saline. BLES (BLES Biochemicals, London, ON, Canada) is a commercially available clinical preparation, stored in 100 mM sodium chloride and 1.5 mM calcium chloride with a phospholipid concentration of 27 mg/ml and contains all-natural phospholipids found in bovine surfactant, along with surfactant-associated proteins SP-B and SP-C. BLES (re-suspended in sterile saline) and peptide were mixed to concentrations of 10 mg/ml phospholipid of BLES, and 0–200 µM CATH-2.

### Clinical CF Sputum Isolates

Clinical isolates were obtained from ten adult CF patients attending the London Health Sciences Centre outpatient CF clinics and protocols were approved by the Human Ethics subcommittee at Western University, London, ON, Canada. Informed consent was obtained from all patients participating in the study. All methods were performed in accordance with the Canadian Institutes of Health Research Tri-council Policy Statement: *Ethical Conduct for Research Involving Humans*, December 2010. Isolated bacteria were stored in citrulline solution at −80 °C, and were isolated, identified and sub-cultured onto chocolate blood agar (CBA) prior to use in study. The samples obtained included seven *Pseudomonas aeruginosa*, one *Achromobacter xylosoxidans*, and three *Staphylococcus aureus* bacterial strains isolated from CF sputum samples. Antibiotic susceptibility profiles of the isolated bacteria were obtained usingVitek-2 ID/AST Instrument (bioMérieux, France).

### Bactericidal activity against clinical isolates

Bactericidal activity was measured using a spot plating assay. In brief, an overnight culture of bacteria grown in TSB diluted in Mueller-Hinton Broth (MHB). The turbidity was adjusted to 0.5 McFarland, followed by an additional 50x dilution. Subsequently, 50 µL of bacteria and 50 µL of either CATH-2 or BLES+CATH-2 was added to a polypropylene coated 96-well plate, resulting in a concentration range of CATH-2 from 0–100 µM with or without 10 mg/ml BLES. These bacterial suspensions were incubated at 37 °C for three hours with no shaking after which they were serially diluted 10–10 000-fold and 10 µL of each dilution was spot plated in triplicate on CBA plates. These plates were incubated overnight, and colonies on the plates were counted the following morning. Minimum bactericidal concentration (MBC) was determined as the minimum CATH-2 concentration at which no bacterial growth was observed at the highest dilution, corresponding to a bacterial concentration of less than 100 CFU/ml.

### Anti-inflammatory effects against clinical isolates

One *P. aeruginosa* and one *S. aureus* species were randomly selected for use in this experiment. An overnight culture of *P. aeruginosa* or *S. aureus* was diluted 1/10 in MHB. The optical density was measured using a spectrophotometer, and bacteria were further diluted in MHB to reach an initial concentration of approximately 2 × 10^6^ CFU/ml. Subsequently, the bacteria were killed by heat (90 °C for one hour) with complete bacterial killing confirmed via spot plating on chocolate agar and incubated overnight to assess for bacterial growth. In a subset of bacteria killed by heat, CATH-2 (20 µM) or BLES+CATH-2 (10 mg/ml phospholipid; 100 µM CATH-2) was added to the killed-bacteria. Thereafter, the dead-bacterial suspensions were the instilled into male C57Bl/6 mice (Charles River, Sherbrooke, Qc, Canada) as previously described^40^. All methods were carried out in accordance with guidelines and regulations set forth by the Western University Council for Animal Care, the Canadian Council on Animal Care and Ontario Animals in Research Act. Briefly, once intubated, mice were randomized to one of 4 experimental groups with all receiving a 50 µL instillation of either: 1) heat-killed bacteria, 2) heat-killed bacteria supplemented with CATH-2, 3) heat-killed bacteria supplemented with BLES+CATH-2, or 4) control MHB without bacteria. Mice were subsequently extubated and allowed to recover and breathe spontaneously for six hours. Following the recovery period, mice were euthanized by sodium pentobarbital overdose and aortic transection, and were placed on a FlexiVent© to measure quasi-static lung compliance and elastance. Following Flexivent measurements, whole lung bronchoalveolar lavage fluid (BALF) was collected by flushing the lungs with 3 aliquots of 1 ml sterile saline. The whole lung lavage was immediately centrifuged at 400 x g at 4 °C, and the pellet was collected for cell analysis, while the supernatant was collected and used to measure protein content and cytokine quantification.

Cell counts and differential cell analysis of the cell pellets obtained from the lavage was done as previously described^61^. After centrifugation, the cell pellet was resuspended in Plasma-Lyte A solution (Baxter Healthcare, Dearfield, IL), and were visualized with 1:1 v/v of 0.4% trypan blue for cell counts via a Bright-Line hemocytometer (Hausser Scientific, Horsham, PA). For cell differential analysis, the cell pellet was centrifuged at 1000 rpm using a Shandon Cytospin 4 (Thermo Scientific, Cheshire, UK) for six minutes, and were fixed using a Hemacolor hematoxylin/eosin kit according to manufacturer’s instructions (EM Science, Gibbstown, NJ). Protein content of the lavage fluid was measured using a Micro BCA protein assay kit (Pierce, Rockford, Ill., USA), per manufacturer’s instructions. Concentrations of mouse cytokines were measured using multiplexed immunoassay kits per manufacturers’ instructions (R&D Systems, Minneapolis, MN). A Bio-Plex 200 readout system was used (Bio-Rad), which utilizes Luminex® xMAP fluorescent bead-based technology (Luminex Corporation, Austin, TX). Cytokine levels (pg/mL) were automatically calculated from standard curves using Bio-Plex Manager software (v. 4.1.1, Bio-Rad).

To further explore the anti-inflammatory effects of BLES+CATH-2, a second experiment was performed with a laboratory strain of *P. aeruginosa* (ATCC 27853). The goal of this experiment was to test if co-administration of CATH-2 or BLES+CATH-2 was essential to provide its anti-inflammatory effects against heat-killed *P. aeruginosa*. Experimental procedures were similar as described above with the following modifications. Once intubated, animals received two intratracheal instillations of 50 μL. The first suspension was instilled into the lungs via the endotracheal tube. After 15 minutes, 50 μL of the second suspension was instilled, followed by extubation and subsequent intraperitoneal injection of atipamezole hydrochloride (1 mg/kg BW; Zoetis Canada), a dexmedetomidine-counteracting agent. The eight experimental groups, with in brackets the 1^st^ and 2^nd^ installation respectively, were: 1. Negative Control (1^st^: Saline, 2^nd^: Saline), 2. Positive Control (1^st^: Heat killed *P. aeruginosa*, 2^nd^: Saline), 3. Co-administered BLES (1^st^: Heat killed *P. aeruginosa*+BLES, 2^nd^: Saline) 4. Delayed-administered BLES (1^st^: Heat killed P. aeruginosa, 2^nd^: BLES), 5. Co-administered CATH-2 (1^st^: Heat killed *P. aeruginosa*+CATH-2, 2^nd^: Saline) 6. Delayed-administered CATH-2, (1^st^: Heat killed *P. aeruginosa*, 2^nd^: CATH-2) 7. Co-administered BLES+CATH-2 (1^st^: Heat killed *P. aeruginosa* BLES+CATH-2, 2^nd^: Saline), 8. Delayed-administered BLES+CATH-2 (1^st^: Heat killed *P. aeruginosa*, 2^nd^: BLES+CATH-2). The installation was followed by a 4-hour recovery period after which the mice were euthanized, the lungs were lavaged and the lavage fluid was analyzed for differential cell analysis and cytokine concentrations by ELISA. The TNF-α and IL-6 cytokines were analyzed using a BD OptEIA ELISA (BD Biosciences, San Diego, CA), while KC was analyzed using a DuoSet ELISA (R&D Systems, Minneapolis, MN).

### Statistical Analysis

All data generated during this study are included in this published article. Data from the *in vitro* killing assays represent the average of at least three independent repetitions. Results from the *in vivo* experiments were obtained with n-values of 6–8 mice per group. Statistical significance was determined by two way analysis of variance and one-way analysis of variance (ANOVA) followed by a Dunett’s post hoc test to elucidate differences versus positive and negative control values and a Tukey’s post hoc test to determine differences among experimental groups. Results are presented as mean ± the standard error of the mean (SEM), and were considered statistically significant with a P-value of less than 0.05.

### Data Availability

The datasets generated during and/or analysed during the current study are available from the corresponding author on reasonable request.

## References

[1] Stoltz DA, Meyerholz DK, Welsh MJ (2015). Origins of Cystic Fibrosis Lung Disease. N. Engl. J. Med..

[2] Pezzulo AA (2012). Reduced airway surface pH impairs bacterial killing in the porcine cystic fibrosis lung. Nature.

[3] Waters V, Smyth A (2015). Cystic fibrosis microbiology: Advances in antimicrobial therapy. J. Cyst. Fibros..

[4] Hewer SL (2012). Inhaled antibiotics in cystic fibrosis: what’s new?. J. R. Soc. Med..

[5] Merlo CA (2007). Incidence and risk factors for multiple antibiotic-resistant Pseudomonas aeruginosa in cystic fibrosis. Chest.

[6] Marcos, V. *et al*. Free DNA in cystic fibrosis airway fluids correlates with airflow obstruction. *Mediators Inflamm*. **2015**, 10.1155/2015/408935 (2015). 10.1155/2015/408935 PMC4397025 25918476

[7] Mall M, Grubb BR, Harkema JR, O’Neal WK, Boucher RC (2004). Increased airway epithelial Na+ absorption produces cystic fibrosis-like lung disease in mice. Nat. Med..

[8] Cohen TS, Prince A (2012). Cystic fibrosis: a mucosal immunodeficiency syndrome. Nat. Med..

[9] Yu H, Head NE (2002). Persistent Infections and Immunity in Cystic Fibrosis. Front. Biosci..

[10] Sadikot RT, Blackwell TS, Christman JW, Prince AS (2005). Pathogen-host interactions in pseudomonas aeruginosa pneumonia. Am. J. Respir. Crit. Care Med..

[11] Mizgerd JP (2012). Respiratory infection and the impact of pulmonary immunity on lung health and disease. Am. J. Respir. Crit. Care Med..

[12] Yu H, Hanes M, Chrisp CE, Boucher JC (1998). Microbial Pathogenesis in Cystic Fibrosis: Pulmonary Clearance of Mucoid Pseudomonas aeruginosa and Inflammation in a Mouse Model of Repeated Respiratory Challenge. Infect. Immun..

[13] Hancock REW, Sahl H-G (2006). Antimicrobial and host-defense peptides as new anti-infective therapeutic strategies. Nat. Biotechnol..

[14] Marr AK, Gooderham WJ, Hancock RE (2006). Antibacterial peptides for therapeutic use: obstacles and realistic outlook. Curr. Opin. Pharmacol..

[15] Afacan NJ, Yeung AT, Pena OM, Hancock RE (2012). Therapeutic Potential of Host Defense Peptides in Antibiotic-resistant Infections. Curr. Pharm. Des..

[16] Hancock REW, Haney EF, Gill EE (2016). The immunology of host defence peptides: beyond antimicrobial activity. Nat. Rev. Immunol..

[17] Pompilio A (2012). Potential novel therapeutic strategies in cystic fibrosis: antimicrobial and anti-biofilm activity of natural and designed α-helical peptides against Staphylococcus aureus, Pseudomonas aeruginosa, and Stenotrophomonas maltophilia. BMC Microbiol..

[18] Pompilio A (2011). Antibacterial and anti-biofilm effects of cathelicidin peptides against pathogens isolated from cystic fibrosis patients. Peptides.

[19] Mardirossian M (2016). *In vitro* and *in vivo* evaluation of BMAP-derived peptides for the treatment of cystic fibrosis-related pulmonary infections. Amino Acids.

[20] Bals R, Weiner DJ, Meegalla RL, Wilson JM (1999). Transfer of a cathelicidin peptide antibiotic gene restores bacterial killing in a cystic fibrosis xenograft model. J. Clin. Invest..

[21] Nagant C (2012). Identification of peptides derived from the human antimicrobial peptide LL-37 active against biofilms formed by Pseudomonas aeruginosa using a library of truncated fragments. Antimicrob. Agents Chemother..

[22] Kościuczuk EM (2012). Cathelicidins: family of antimicrobial peptides. A review. Mol. Biol. Rep..

[23] Brogden KA (2005). Antimicrobial peptides: pore formers or metabolic inhibitors in bacteria?. Nat. Rev. Microbiol..

[24] Durr UHN, Sudheendra U, Ramamoorthy A (2006). LL-37, the only human member of the cathelicidin family of antimicrobial peptides. Biochim. Biophys. Acta.

[25] Vandamme D, Landuyt B, Luyten W, Schoofs L (2012). A comprehensive summary of LL-37, the factotum human cathelicidin peptide. Cell. Immunol..

[26] Kharasch VS (1991). Pulmonary surfactant as a vehicle for intratracheal delivery of technetium sulfur colloid and pentamidine in hamster lungs. Am. Rev. Respir. Dis..

[27] Birkun A (2014). Exogenous pulmonary surfactant as a vehicle for antimicrobials: assessment of surfactant-antibacterial interactions *in vitro*. Scientifica (Cairo)..

[28] van’t Veen A, Mouton JW, Gommers D, Lachmann B (1996). Pulmonary surfactant as vehicle for intratracheally instilled tobramycin in mice infected with Klebsiella pneumoniae. Br. J. Pharmacol..

[29] van’t Veen A (1996). Exogenous pulmonary surfactant as a drug delivering agent: influence of antibiotics on surfactant activity. Br. J. Pharmacol..

[30] Wang Y, Walter G, Herting E, Agerberth B, Johansson J (2004). Antibacterial activities of the cathelicidins prophenin (residues 62 to 79) and LL-37 in the presence of a lung surfactant preparation. Antimicrob. Agents Chemother..

[31] Banaschewski BJH (2015). Antimicrobial and biophysical properties of surfactant supplemented with an antimicrobial peptide for treatment of bacterial pneumonia. Antimicrob. Agents Chemother..

[32] Govan JRW, Deretic V (1996). Microbial Pathogenesis in Cystic Fibrosis: Mucoid Pseudomonas aeruginosa and Burkholderia cepacia. Microbiol. Rev..

[33] Gómez MI, Prince A (2007). Opportunistic infections in lung disease: Pseudomonas infections in cystic fibrosis. Curr. Opin. Pharmacol..

[34] Cobb LM, Mychaleckyj JC, Wozniak DJ, López-Boado YS (2004). Pseudomonas aeruginosa flagellin and alginate elicit very distinct gene expression patterns in airway epithelial cells: implications for cystic fibrosis disease. J. Immunol..

[35] Dasenbrook EC, Konstan MW, Lechtzin N, Boyle MP (2010). Association Between Respiratory Tract Methicillin-Resistant Staphylococcus aureus and Survival in Cystic Fibrosis. J. Am. Med. Assoc..

[36] Paranjape SM, Mojayzel PJ (2014). Cystic fibrosis. Pediatr. Rev..

[37] Emerson J, Mcnamara S, Buccat AM, Worrell K, Burns JL (2010). Changes in Cystic Fibrosis Sputum Microbiology in the United States Between 1995 and 2008. Pediatr. Pulmonol..

[38] LiPuma JJ (2010). The changing microbial epidemiology in cystic fibrosis. Clin. Microbiol. Rev..

[39] Coorens, M. *et al*. Cathelicidins inhibit E. coli-induced TLR2 and TLR4 activation in a viability-dependent manner. *J. Immunol*. July, 10.4049/jimmunol.1602164 (2017). 10.4049/jimmunol.1602164 PMC5544931 28710255

[40] Coorens, M. *et al*. Killing of P. aeruginosa by chicken cathelicidin-2 prevents lung inflammation *in vivo* No Title. *Infect. Immun*. Submitted (2017). 10.1128/IAI.00546-17 PMC5695126 28947647

[41] Montgomery GS, Howenstine M (2009). Cystic Fibrosis. Pediatr. Rev..

[42] Kapetanovic R (2011). Mechanisms of TNF induction by heat-killed Staphylococcus aureus differ upon the origin of mononuclear phagocytes. AJP Cell Physiol..

[43] van’t Veen A (1995). Influence of pulmonary surfactant on *in vitro* bactericidal activities of amoxicillin, ceftazidime, and tobramycin. Antimicrob. Agents Chemother..

[44] van’t Veen A (1999). Lung clearance of intratracheally instilled 99mTc-tobramycin using pulmonary surfactant as vehicle. Br. J. Pharmacol..

[45] Haitsma JJ, Lachmann U, Lachmann B (2001). Exogenous surfactant as a drug delivery agent. Advanved Drug Deliv. Rev..

[46] Gommers D, Haitsma JJ, Lachmann B (2006). Surfactant as a carrier: influence of immunosuppressive agents on surfactant activity. Clin. Physiol. Funct. Imaging.

[47] Lewis JF, McCaig L (1993). Aerosolized versus instilled exogenous surfactant in a nonuniform pattern of lung injury. Am. Rev. Respir. Dis..

[48] Duplantier AJ, van Hoek ML (2013). The human cathelicidin antimicrobial peptide LL-37 as a potential treatment for polymicrobial infected wounds. Front. Immunol..

[49] Bommineni YR (2010). A fowlicidin-1 analog protects mice from lethal infections induced by methicillin-resistant Staphylococcus aureus. Peptides.

[50] Schneider VAF (2016). Imaging the antimicrobial mechanism(s) of cathelicidin-2. Sci. Rep..

[51] Coorens M, Scheenstra MR, Veldhuizen EJA, Haagsman HP (2017). Interspecies cathelicidin comparison reveals divergence in antimicrobial activity, TLR modulation, chemokine induction and regulation of phagocytosis. Sci. Rep..

[52] Subramanian H, Gupta K, Guo Q, Price R, Ali H (2011). Mas-related gene ×2 (MrgX2) is a novel G protein-coupled receptor for the antimicrobial peptide LL-37 in human mast cells: Resistance to receptor phosphorylation, desensitization, and internalization. J. Biol. Chem..

[53] Mader JS, Marcet-Palacios M, Hancock REW, Bleackley RC (2011). The human cathelicidin, LL-37, induces granzyme-mediated apoptosis in cytotoxic T lymphocytes. Exp. Cell Res..

[54] Van Dijk, A. *et al*. Immunomodulatory and anti-inflammatory activities of chicken cathelicidin-2 derived peptides. *PLoS One* **11** (2016). 10.1371/journal.pone.0147919 PMC4743981 26848845

[55] van Dijk A (2009). Identification of chicken cathelicidin-2 core elements involved in antibacterial and immunomodulatory activities. Mol. Immunol..

[56] Xiao Y (2009). The central kink region of fowlicidin-2, an alpha-helical host defense peptide, is critically involved in bacterial killing and endotoxin neutralization. J. Innate Immun..

[57] Veldhuizen EJA (2017). Antimicrobial and immunomodulatory activity of PMAP-23 derived peptides. Protein Pept. Lett..

[58] Torres A (2015). Bacteraemia and antibiotic-resistant pathogens in community acquired pneumonia: risk and prognosis. Eur. Respir. J..

[59] Micek ST (2015). An international multicenter retrospective study of Pseudomonas aeruginosa nosocomial pneumonia: Impact of multidrug resistance. Crit. Care.

[60] Bikker FJ (2006). Evaluation of the antibacterial spectrum of drosocin analogues. Chem. Biol. Drug Des..

[61] Walker MG (2009). Elevated endogenous surfactant reduces inflammation in an acute lung injury model. Exp. Lung Res..

